# Mobile dating app use and sexual risk behavior among Brazilian undergraduate students

**DOI:** 10.47626/2237-6089-2023-0746

**Published:** 2025-04-07

**Authors:** Edson Zangiacomi Martinez, Vitoria de Souza Pinto Frazatto, Jonathan Leonardo Gonçalves Prudencio, Guilherme Galdino, Miriane Lucindo Zucoloto

**Affiliations:** 1 Universidade de São Paulo Faculdade de Medicina de Ribeirão Preto Departamento de Medicina Social Ribeirão Preto SP Brazil Departamento de Medicina Social, Faculdade de Medicina de Ribeirão Preto, Universidade de São Paulo (USP), Ribeirão Preto, SP, Brazil.

**Keywords:** Sexual behavior, mobile applications, students, health promotion

## Abstract

**Objective:**

One of the most popular ways to meet new people in the modern world is through dating apps. However, their use may facilitate casual sexual encounters and quick partner changes, both of which are associated with endangering sexual health in different populations. The objective of this study is to describe the use of mobile dating apps among undergraduate students at a major Brazilian public university and investigate its associations with sexual risk behaviors and sociodemographic factors.

**Methods:**

This is a cross-sectional study based on a web survey. The link for participation was made available to students enrolled in undergraduate courses at the eight units of Universidade de São Paulo, Campus Ribeirão Preto. Use of dating apps, sociodemographic/behavioral profile, and sexual risk behaviors were among the variables studied. The distribution of app users was calculated for each variable of interest, and prevalence ratios (PRs) were used for comparisons. PRs were reported with 95% confidence intervals (95%CI).

**Results:**

A total of 487 students participated, with 32.9% reporting using dating apps. Male participants were more likely to use apps. Use of dating apps was associated with having multiple sexual partners and risky behaviors such as smoking, alcohol consumption, and substance abuse.

**Conclusion:**

It is critical to describe the pattern of app use by undergraduate students and understand its influence on sexual health in order to avoid stigmatizing users. Additionally, this information can be helpful to guide the creation of strategies for using these apps as resources to promote health, such as through sharing information regarding sexual health.

## Introduction

Mobile dating apps are one of the most popular ways to meet new people in the modern world, having been used by millions of users. The majority of dating apps, including Tinder – the most popular such app in Brazil – are free to download and use, and can be viewed on any smartphone or other internet-connected device.

According to Albury and Byron^1^ and Anzani et al.,^2^ dating apps offer significant advantages for interpersonal connection, including the chance to vet potential partners before a date, explore sexual identity, meet more partners in geographical proximity, and decide how and when to connect with them. These advantages grew during the COVID-19 pandemic, as dating apps introduced new virtual-dating options to adhere to physical distancing guidelines.^3-5^

Beyond these advantages, however, using dating apps can also encourage greater exposure to risk behaviors according to recent publications.^6-9^ Studies have linked use of dating apps to risky behaviors such as having multiple sexual partners, since their use may facilitate casual sexual encounters and rapid partner change, with a higher chance of having unprotected sex, which may endanger sexual health.^10,11^

Young people are the most likely to use new technology to connect with others for friendship, dating, and sexual relationships.^12^ However, few studies have been conducted to investigate and generate evidence regarding the relationship between using dating apps and the sexual health of youths of various sexual orientations.^8,9,11,12^ The majority of previous studies have concentrated on the experiences of homosexual men; it is unclear how using dating apps affects the sexual health of other populations.^13-15^

In this sense, this study aims to describe the use of dating apps among undergraduate students at a major Brazilian public university, examining its associations with sexual risk behaviors and sociodemographic and behavioral factors, including age, gender, sexual orientation, relationship status, monthly income, and smoking and drinking habits.

## Methods

This cross-sectional study was conducted on a sample of undergraduate students enrolled at the Ribeirão Preto campus of the Universidade de São Paulo (USP-RP), located in Southeast Brazil. At the time of the survey, the campus had a total of 7,181 undergraduate students enrolled. The sampling process involved a stratified design that considered each of the eight academic units on the campus as a separate stratum. Data collection occurred between March and May 2021, a period during which social isolation measures were in effect in Brazil due to the coronavirus disease 2019 (COVID-19) pandemic. To minimize personal contact, an online self-administered questionnaire was employed for data collection via the REDCap platform. The study was promoted in collaboration with academic centers, student leagues, and athletic associations at USP-RP, who helped publicize the research among students within each stratum through various social media channels such as Facebook groups, WhatsApp, and e-mail.

The authors developed the study questionnaire by reviewing existing literature and drawing on prior studies by the same group.^8,9,15^ The online survey comprised three sections: use of dating apps, sexual behavior, and sociodemographic and behavioral profile. The following question regarding use of apps was posed to participants: "Have you ever used mobile applications or websites to search for sexual partners?" Additionally, some sociodemographic and behavioral questions were posed, including items related to age, gender, sexual orientation, relationship status, monthly income, and smoking and drinking habits. Questions about sexual relations with occasional partners in the previous 12 months (male and female), the number of these partners, and condom use were used to assess risk behavior.

The distribution of app users was calculated for each variable of interest, and prevalence ratios (PRs) were used for comparisons. PRs were reported with 95% confidence intervals (95%CI). CIs that do not include the value 1 indicate significant associations (similar to p < 0.05). Data were organized and analyzed using R statistical software (version 4.1.0, www.r-project.org/).

### Ethical considerations

The study received approval from the local Research Ethics Committee, registered under CAAE number: 31049220.4.0000.5440. An informed consent form was provided on the initial page of the online questionnaire.

## Results

A total of 525 students voluntarily completed the questionnaire, with proportional representation from each of the eight units at USP-RP. However, 38 participants did not answer all of the questions on the questionnaire and were therefore excluded from the final analyses. The final sample comprised 487 students aged between 18 and 46 years (mean 22.2, standard deviation [SD] 3.1 years), with 68.9% identifying as female. Among the male respondents, 60.1% identified themselves as heterosexual, 20.7% as homosexual, and 16% as bisexual. Among the female respondents, 63.2% identified themselves as heterosexual, 5.7% as homosexual, and 27.1% as bisexual.

Use of dating apps was reported by 32.9% of the students. Among these, 47.9% of male participants and 20.7% of female participants reported using Tinder. Grindr was used by 41.2 and 60.3% of partnered and unpartnered male non-heterosexual respondents, respectively. Other apps and websites mentioned by respondents included Hornet, Bumble, Fem Dating, Happn, Instagram, OkCupid, Scruff, Umatch, and Zoe.

Table 1 describes use of dating apps based on participants’ characteristics. Because the frequency of app use among female and male participants is moderated by relationship status and sexual orientation, we used combinations of the classes of these variables in Table 1. Among our results, older participants, non-heterosexual men (partnered and unpartnered), unpartnered heterosexual men, those who had previously engaged in sexual activity, those with a history of sexually transmitted infections, those who were currently using alcohol or tobacco, those who reported the use of drugs, and those who were financially independent were more likely to use dating apps.

**Table 1:** Use of dating apps according to participants’ characteristics (n = 487), Ribeirão Preto, São Paulo, Brazil, 2021

		Total*	App users n (%)	PR (95%CI)
Age (years)				
	18- 20	86	19 (22.1)	Ref.
	20- 22	132	41 (31.1)	1.41 (0.88-2.25)
	22- 24	101	38 (37.6)	1.70 (1.06-2.72†)
	24- 26	42	16 (38.1)	1.72 (0.99-3.00)
	26 or more	34	19 (55.9)	2.53 (1.54-4.16†)
				
Relationship status, sex, and sexual orientation				
	Partnered female heterosexual	94	6 (6.4)	Ref.
	Partnered male heterosexual	53	9 (17.0)	2.66 (1.00-7.05)
	Partnered female non-heterosexual	48	11 (22.9)	3.58 (1.41-9.09†)
	Unpartnered female heterosexual	95	22 (23.2)	3.62 (1.54-8.54†)
	Unpartnered female non-heterosexual	62	23 (37.1)	5.80 (2.50-13.42†)
	Unpartnered male heterosexual	60	30 (50.0)	7.81 (3.46-17.64†)
	Partnered male non-heterosexual	17	10 (58.8)	9.19 (3.85-21.94†)
	Unpartnered male non-heterosexual	58	49 (84.5)	13.20 (6.04-28.86†)
				
Ethnicity				
	White	379	124 (32.7)	Ref.
	Brown	61	21 (34.4)	1.05 (0.72-1.53)
	Black	23	7 (30.4)	0.93 (0.49-1.75)
	Yellow	18	6 (33.3)	1.02 (0.52-1.99)
				
Ever had sexual intercourse				
	No	49	7 (14.3)	Ref.
	Yes	430	152 (35.3)	2.47 (1.23-4.96†)
				
Religion				
	Yes	204	47 (23.0)	Ref.
	No, but believes in God	160	53 (33.1)	1.44 (1.03-2.01†)
	Atheist	103	51 (49.5)	2.15 (1.57-2.96†)
				
History of STIs				
	No	439	136 (31.0)	Ref.
	Yes	47	24 (51.1)	1.65 (1.21-2.25†)
				
Smoking status				
	Never smoked	313	83 (26.5)	Ref.
	Current smoker	145	63 (43.4)	1.64 (1.26-2.13†)
	Ex-smoker	17	5 (41.7)	1.57 (0.79-3.15)
				
Current alcohol use				
	No	114	21 (18.4)	Ref.
	Yes	368	137 (37.2)	2.04 (1.35-3.03†)
				
Current drug use				
	No	329	82 (24.9)	Ref.
	Yes	149	75 (50.3)	2.02 (1.58-2.58†)
				
Financially independent				
	No	440	134 (30.5)	Ref.
	Yes	47	26 (55.3)	1.82 (1.35-2.44†)

95%CI = 95% confidence interval; PR = prevalence ratio; STI = sexually transmitted infection.

* Numbers may not add up to total due to missing values.

† 95%CIs that do not include the value 1 show significant associations.

Meanwhile, Table 2 provides information on the sexual behaviors of students who had had occasional partners in the last 12 months, categorized by sex. Among those participants who have occasional partners, males who only had sex with male occasional partners and females who had sex with male and female occasional partners were more likely to use apps.

**Table 2:** Students’ sexual behaviors with occasional partners in the last 12 months, stratified by sex (n = 487), Ribeirão Preto, São Paulo, Brazil, 2021

Sexual behaviors in the last 12 months			Total	App users n (%)	PR (95%CI)
Male					
	Had sex with occasional partners (n = 188)				
		No	110	37 (33.6)	Ref.
		Only male occasional partners	35	32 (91.4)	2.72 (2.05-3.60*)
		Only female occasional partners	37	25 (67.6)	2.01 (1.43-2.84*)
		Both male and female partners	6	4 (66.7)	1.99 (1.06-3.70*)
	Number of male occasional partners (n = 41)				
		Only one	10	7 (70.0)	Ref.
		2-4	21	19 (90.5)	1.29 (0.84-1.99)
		5-10	7	7 (100.0)	1.43 (0.95-2.14)
		>10	3	3 (100.0)	1.43 (0.95-2.14)
	Number of female occasional partners (n = 43)				
		Only one	13	8 (61.5)	Ref.
		2-4	17	11 (64.7)	1.05 (0.60-1.83)
		5-10	10	7 (70.0)	1.14 (0.63-2.06)
		>10	3	3 (100.0)	1.63 (1.06-2.50*)
	Condom use with male occasional partners (n = 41)				
		Always used	19	18 (94.7)	Ref.
		Used most of the time	11	10 (90.9)	0.96 (0.77-1.19)
		Not used most of the time	3	3 (100.0)	1.06 (0.95-1.17)
		There was no penetration	6	5 (83.3)	-
		Not used at all	1	0 (0.0)	-
	Condom use with female occasional partners (n = 43)				
		Always used	26	15 (57.7)	Ref.
		Used most of the time	8	6 (75.0)	1.30 (0.77-2.18)
		Not used most of the time	7	7 (100.0)	1.73 (1.25-2.41*)
		There was no penetration	2	1 (50.0)	-
Female					
	Had sex with occasional partners				
		No	211	26 (12.3)	Ref.
		Only male occasional partners	68	27 (39.7)	3.23 (2.03-5.13*)
		Only female occasional partners	7	3 (42.9)	3.49 (1.38-8.82*)
		Both male and female partners	12	6 (50.0)	4.07 (2.08-7.95*)
	Number of male occasional partners (n = 80)				
		Only one	22	6 (27.3)	Ref.
		2-4	47	21 (44.7)	1.64 (0.77-3.48)
		5-10	10	5 (50.0)	1.83 (0.73-4.60)
		>10	1	1 (100.0)	3.66 (1.85-7.25*)
	Number of female occasional partners (n = 19)				
		Only one	16	7 (43.8)	Ref.
		2-4	2	1 (50.0)	1.14 (0.26-5.08)
		5-10	1	1 (100.0)	2.28 (1.31-3.98*)
	Condom use with male occasional partners (n = 80)				
		Always used	36	14 (38.9)	Ref.
		Used most of the time	32	16 (50.0)	1.29 (0.75-2.20)
		Not used most of the time	10	3 (30.0)	0.77 (0.27-2.16)
		There was no penetration	1	0 (0.0)	-

95%CI = 95% confidence interval; PR = prevalence ratio.

* CIs that do not include the value 1 show significant associations.

## Discussion

This study found that 32.9% of the students reported using dating apps. This percentage is lower than that found in a previous study that enrolled students from a university in the Brazilian state of Tocantins, in the Legal Amazon (66.3%).^16^ Another study, which included freshmen undergraduate students at a university in the southern region of Brazil, found that 22% of them had used smartphone apps to seek sex within 3 months prior to the survey.^17^ While there are few studies published in Brazil on the use of apps by undergraduate students, these results suggest that the prevalence of usage may be influenced by regional differences and population profiles.

According to a literature review, the average dating app user profile is characterized by white men who have sex with men (MSM), aged 25 to 35, with high education and income, who have frequent sexual encounters, and often engage in risky behavior.^2,18^ In our study, we did not find an association between app use and students’ ethnicity. Nonetheless, we observed a higher prevalence of dating app users among those over 25 years of age, sexually active individuals, those who identify as atheist or non-religious, those who are financially independent, those with a history of sexually transmitted infections, smokers, and individuals who use drugs or alcohol. When examining the interaction between relationship status, sex, and sexual orientation, the results show that dating app use is relatively low among partnered heterosexual female students (6.4%), but significantly higher among partnered and unpartnered male non-heterosexual students (58.8 and 84.5%, respectively).

A considerable amount of research on app use by MSM has been carried out worldwide.^13-15^ Most of these studies describe MSM engaging in high-risk behaviors and state that apps can offer opportunities for providing information about sexual health and HIV prevention, and for promoting HIV testing.^19^ In their findings about the acceptability and potential impact of delivering sexual health promotion information via social media and dating apps, Kesten et al.^20^ suggested that these tools appear to be acceptable and effective ways to reach key populations. They also emphasize the importance of such information being engaging, simple, not overly clinical, and focused on developing preventative social norms.

In our study, men and women who have relationships with occasional partners, both in homosexual and heterosexual relationships, often use dating apps. Use of condoms during sexual encounters varies among different groups. For men who had occasional same-sex partners, 73.2% reported using condoms all or most of the time. For men who had occasional sex with women, 79.1% reported using condoms all or most of the time. The percentage of condom users was highest among women who had sex with occasional male partners, with 85% reporting using condoms all or most of the time. In all of these situations, however, our study did not show any clear association between use of dating apps and condom use.

Tinder was the most commonly used dating app among the sample. However, male non-heterosexual students also reported using Grindr and Hornet, which are specifically designed for gay individuals. Some websites have rated OkCupid as better for more serious dating and Tinder for more casual dating,^10^ although some surveys show that many Tinder users use the app to look for romantic relationships.^6,18^ In addition, with respect to other dating apps, a literature review showed that the Tinder use is less related to the risk of sexually transmitted infections (STIs).^6^ Irrespective of the dating app being used and the sexual orientation of the user, this study shows associations between dating app usage and history of STIs and risky behaviors such as smoking, alcohol consumption, and substance abuse. Although our findings do not show any association with inappropriate use of condoms, our results reinforce that dating apps can offer opportunities for providing information about sexual health and STI prevention, and for promoting HIV testing.^19^ Given that the relationship between dating app use and sexual risk behaviors is also present among heterosexual young adults,^21^ opportunities to provide sexual health and risk education through the apps can be considered for young people of all sexual orientations.

This study has some limitations, including the fact that the data were collected during the COVID-19 pandemic, when individuals were required to stay at home and limit their social interactions, which also precluded face-to-face interviews. The data for this study were collected from a sample of college students in the southeastern region of Brazil. Therefore, care should be taken when extending the findings to other populations and geographic areas. Additionally, the self-reported nature of the data, as well as the possibility of self-selection bias within strata, may affect the accuracy and generalizability of the results. Despite these limitations, this study is one of the few to have assessed the relationship between use of mobile dating apps and sexual and risky sexual behaviors in Brazilian young adults, not restricted to the homosexual population. The findings describe the popularity of dating apps among college students and their motivations for using them and suggest that these apps may offer opportunities to provide sexual health education to young adults.

## Conclusion

Our study found that use of dating apps among undergraduate students was common and presents evidence of associations between dating app usage and history of STIs and risky behaviors such as smoking, alcohol consumption, and substance abuse. Despite these findings, and the fact that numerous studies have suggested that dating apps have a negative impact on sexual health, it is important to recognize that they are widely used and should not be stigmatized. In fact, dating apps have the potential to be effective resources for promoting health.
